# Imaging of Primitive Neuroectodermal Tumor (PNET) by Technetium-99m Tetrofosmin

**DOI:** 10.1080/13577140120099218

**Published:** 2001-12

**Authors:** Georgios Karanikas, Wolfgang Köstler, Alexander Becherer, Robert Dudczak, Michael Krainer, Kurt Kletter

**Affiliations:** 1 Departments of Nuclear Medicine and Internal Medicine I University of Vienna Vienna Austria; 2 Division of Oncology University of Vienna Vienna Austria; 3 Department of Nuclear Medicine University of Vienna Waehringer Guertel 18–20 Vienna A–1090 Austria

## Abstract

*Purpose.* Tc-99m tetrofosmin launched for myocardial studies has recently also shown a good detectability for several tumors. Data on PNET imaging by Tc-99m tetrofosmin are not yet available.

*Patient and method.* In the case of a 21-year-old man suffering from pelvis PNET, Tc-99m tetrofosmin scintigraphy was performed additionally to CT and MRI.

*Results.* The gluteal and iliac tumor masses were visualized by Tc-99m tetrofosmin according to the CT and MRI results.

*Discussion.* Imaging with Tc-99m tetrofosmin could provide additional information to the available conventional radiological imaging modalities for diagnosis of PNET, and could be a useful tool for the restaging of the primary tumor.


*Sarcoma* (2001) 5, 215–216

CASE REPORT

### Imaging of primitive neuroectodermal tumor (PNET) by technetium-99m tetrofosmin

GEORGIOS KARANIKAS[1], WOLFGANG KÖSTLER[2], ALEXANDER BECHERER[1], ROBERT DUDCZAK[1], MICHAEL KRAINER[2] & KURT KLETTER[1]

*Departments of [1]Nuclear Medicine and Internal Medicine I, and [2]Division of Oncology, University of Vienna, Vienna, Austria*

**Abstract**
*Purpose.* Tc-99m tetrofosmin launched for myocardial studies has recently also shown a good detectability for several tumors. Data on PNET imaging by Tc-99m tetrofosmin are not yet available.
*Patient and method.* In the case of a 21-year-old man suffering from pelvis PNET, Tc-99m tetrofosmin scintigraphy was performed additionally to CT and MRI.
*Results.* The gluteal and iliac tumor masses were visualized by Tc-99m tetrofosmin according to the CT and MRI results.
*Discussion.* Imaging with Tc-99m tetrofosmin could provide additional information to the available conventional radiological imaging modalities for diagnosis of PNET, and could be a useful tool for the restaging of the primary tumor.

**Key words:** *PNET, Tc-99m tetrofosmin*

### Patient

In October 1999 a 21-year-old man presented with a painful mass in the right gluteal region which had developed within the last 4 weeks. Previous medical history was without any major events except for lower back pain persisting for the last 12 months despite conservative treatment.

Physical examination revealed a subfebrile patient with a firm, egg-sized right gluteal mass, painful loss of motion in the right hip joint and paresthesia on the ventral and medial part of the right femur. Aside from leukocytosis and an elevated C-reactive protein, laboratory testing was unremarkable.

A pelvic CT scan showed a spindle-shaped mass in the right greatest gluteal muscle and a second mass originating in the right iliac muscle displacing the right ureter and psoas muscle. In addition, hepatomegaly was found, and osteomyelitis of the right iliac bone was suspected. Pathohistological examination of a consecutive incisional biopsy of the gluteal mass diagnosed a primitive neuroectodermal tumor (PNET). Staging examinations, including cranial and thoracic CT scan as well as bone scan, revealed multiple pulmonary lesions (2–5 mm) in the right lower lobe, a left-sided pleural effusion and diffuse infiltration of the lower vertebral spine and pelvis. MRI (Fig. 1) of the pelvis demonstrated that the gluteal and iliac masses were infiltrating the right femur, hip joint, lower lumbar vertebrae and the sacrum with consecutive narrowing of the neuroforamen L5/S1.

### Method and results

### *Technetium-99m tetrofosmin imaging and analysis*

Planar and single photon emission tomography (SPECT) acquisitions were performed with a large

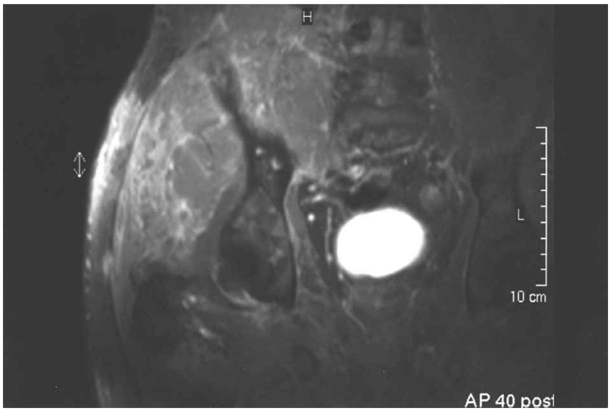

**Fig. 1.** *Enhanced T1 SE sequence shows inhomogeneous enhancement of the tumor in the gluteal area (right side); the bladder is filled with contrast agent.*

Correspondence to: Dr. Georgios Karanikas, Department of Nuclear Medicine, University of Vienna, Waehringer Guertel 18–20, A–1090 Vienna, Austria. Tel: +44-1-404005550. Fax: +43-1-40405532. E-mail: georg.karanikas@akh-wien.ac.at

1357–714X print/1369–1643 online/01/040215–02 © 2001 Taylor & Francis Ltd
DOI: 10.1080/13577140120099218

field-of-view double-headed gamma camera (Siemens ICON) equipped with a low-energy, parallel-hole collimator. Imaging was done 10 min after administration of 580 MBq technetium-99m tetrofosmin. SPECT imaging of the pelvic region was performed subsequently after the planar acquisition.

Scintigraphic data were reconstructed after prefiltering with a low pass filter using filtered backprojection and a ramp filter. Final evaluation of all scans was done from coronal, sagittal and transaxial slices. Evaluation was performed independently by two nuclear medicine physicians with experience in the interpretation of tetrofosmin studies.

Augmented technetium-99m tetrofosmin accumulation was obtained in the left pelvic and hip region (Fig. 2). The uptake of the tracer correlated well with the gluteal and iliac masses as described by MRI (Fig. 3.).

The patient was assigned to receive two cycles of chemotherapy according to the Euro-Ewing protocol, in the course of which he repeatedly developed febrile neutropenia and renal failure which were controlled by haematopoetic growth factors, antibiotics and diuretics. In January 2000 restaging revealed a marked progression of both, pulmonary and pelvic lesions and a new lesion in the thoracic spine compressing the right neuroforamen T7/T8. Subsequently, the patient was assigned to receive palliative irradiation of the thoracic and lumbar spine and the right pelvis. However, his clinical status deteriorated rapidly and 2 weeks later, in February 2000, our patient died due to his underlying disease.

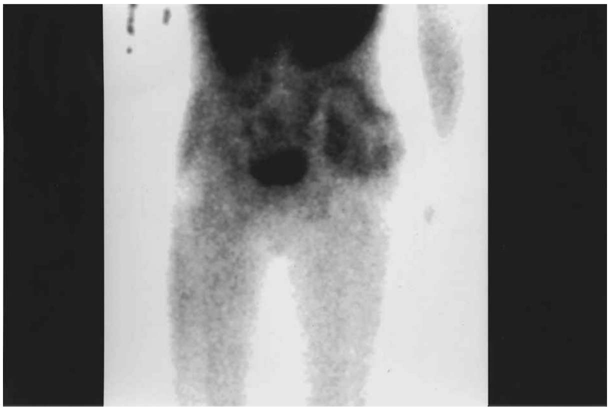

**Fig. 2.** *The technetium-99m tetrofosmin scintigraphy (in posterior view) reveals gluteal and iliac tumor masses in good correlation with MRI. Physiological accumulation of the tracer in gastrointestinal tract and bladder.*

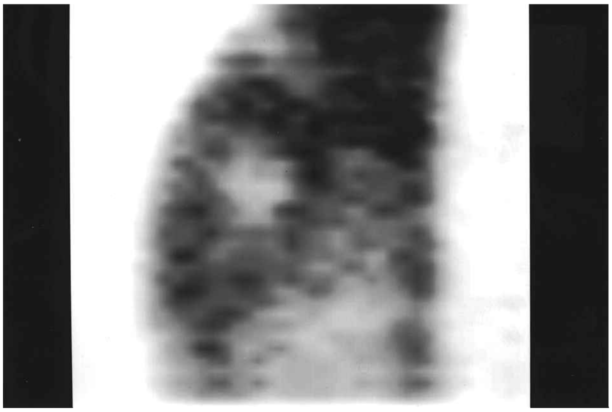

**Fig 3.** *The SPECT image (sagittal) shows the tumor with the necrotic masses.*

### Discussion

Various radiopharmaceuticals have been explored for use in detection and characterization of musculoskeletal sarcomas. The choice of an adequate tumor-depicting radiopharmaceutical in the clinical situation is not always obvious in the expanding field of nuclear oncology. Tetrofosmin, a lipophilic cationic diphosphine, originally developed for myocardial perfusion imaging, has proved to be an excellent tumor-imaging agent, accumulating in thyroid and breast cancers, and recently in a variety of tumors, showing a sensitivity of 95% and above, as well as a quite high specificity.[1–3] Recently, technetium-99m tetrofosmin uptake by several musculoskeletal sarcomas of the extremities or pelvis has been examined.[4] In our case report, a primitive neuroectodermal tumor (PNET) was imaged by technetium-99m tetrofosmin, giving a good correlation with other imaging modalities, such as CT and MRI. Lung metastases were not visualized, probably because of their small size. PNET imaging by Tc-99m tetrofosmin could provide additional information in the staging process and give, furthermore, valuable information in the restaging process of the tumor.

### References

1. Higley B, Smith FW, Smith T, Gemmel HG, Das-Gupta P, Gvozdanovic Dv. Technetium-99m-1,2-bis[bis(2-ethoxyethyl) phosphino]ethane: human biodistribution, dosimetry and safety of a new myocardial perfusion imaging agent. *J Nucl Med* 1993; 34: 30–8.
2. Lind P, Gallowitsch HJ, Langsteger W, Kresnik E, Mikosch P, Gomez I. Technetium-99m-Tetrofosmin whole body scintigraphy in the follow-up of differentiated thyroid carcinoma. *J Nucl Med* 1997; 38: 348–52.
3. Fenlon HM, Phelan NC, Sullivan P, Tierney S, Gorey T, Ennis JT. Benign versus malignant breast disease: comparison of contrast-enhanced MR imaging and Tc-99m tetrofosmin scintimammography. *Radiology* 1997; 205: 214–20.
4. Söderlund V, Jonsson C, Bauer HCF, Brosjö O, Jacobsson H. Comparison of technetium-99m-MIBI and technetium-99m-tetrofosmin uptake by musculoskeletal sarcomas. *J Nucl Med* 1997; 38: 682–6.
